# Ten-year trends of delayed sequential bilateral cataract surgery (DSBCS) in Sweden: a register-based study

**DOI:** 10.1186/s40662-024-00406-0

**Published:** 2024-10-01

**Authors:** Andreas Viberg, Tomas Bro, Anders Behndig, Maria Kugelberg, Madeleine Zetterberg, Ingela Nilsson, Mats Lundström

**Affiliations:** 1 Department of Clinical Sciences, Ophthalmology, Umeå University, 901 85 Umeå, Sweden; 2 Department of Biomedical and Clinical Sciences, Linköping University, Linköping, Sweden; 3 Department of Clinical Neuroscience, Karolinska Institutet, Stockholm, Sweden; 4 Department of Clinical Neuroscience, Sahlgrenska Academy, University of Gothenburg, Mölndal, Sweden; 5 Capio Medocular AB, Malmö, Sweden; 6 Department of Clinical Sciences, Ophthalmology, Faculty of Medicine, Lund University, Lund, Sweden

**Keywords:** Delayed sequential bilateral cataract surgery, Cataract surgery, Cataract, Register-based study

## Abstract

**Purpose:**

To study the trend of delayed sequential bilateral cataract surgery (DSBCS) in Sweden in the past decade.

**Methods:**

This register-based cohort study utilized data from the Swedish National Cataract Register (NCR) from 2010 through 2019. Register files from patients who underwent cataract surgery in both eyes during the study period were linked using their social security numbers. Bilateral surgeries on different days were classified as DSBCS. The study investigated the association between DSBCS within 3 months and several variables with stratification and multivariate logistic regression. The following variables were used: operation year, region, private or public unit, age, sex, indication for surgery, type of intraocular lens (IOL), preoperative visual acuity, ocular comorbidity, posterior capsule rupture and perioperative difficulties.

**Results:**

During the study period, 368,106 patients underwent DSBCS, of which 62.6% (n = 230,331) had bilateral surgery within 3 months. The median time between the surgeries was 61 days (interquartile range 26–161 days), showing regional variations. Better visual acuity in the fellow eye, presence of ocular comorbidity, various perioperative events and complications were associated with longer time to surgery of the second eye. Conversely, cataract surgery in more recent years, private clinic, increasing age, anisometropia and multifocal IOL were associated with shorter timespan between surgeries.

**Conclusions:**

The majority of DSBCS were conducted within a 3-month timeframe, with the interval between surgeries decreasing throughout the study period. Several rational factors were associated with the time difference, in addition to regional variations. Many patients would probably benefit from less time between the surgeries, and we encourage a clinical practice taking the whole patient’s visual function into account.

## Background

Cataract is the most common reversible cause of visual impairment worldwide and affects both eyes in most cases [1]. It is common to have cataract surgery in both eyes but at separate dates; this is commonly known as delayed sequential bilateral cataract surgery (DSBCS) [2, 3].

When both eyes undergo cataract surgery on the same day but as separate procedures it is termed immediate sequential bilateral cataract surgery (ISBCS), which has been shown to be economically beneficial [4, 5]. The extent of ISBCS globally is unknown, as the established metrics overlook this practice [6]. For example, the Organization for Economic Cooperation and Development (OECD) only counts ISBCS as one procedure but DSBCS as two [7]. Concerns about the potential risks of simultaneous bilateral infection/endophthalmitis have impeded widespread acceptance of ISBCS together with governmental regulations, medicolegal implications, economic disincentives, and malpractice insurance coverage regulations. On the other hand, proponents argue that strict adherence to recommended protocols mitigates this risk. Other benefits are the reduction of a growing backlog of treatable blindness and faster visual recovery [8]. In a number studies, no significant difference in postoperative visual acuity, refractive error or endophthalmitis risk has been observed between DSBCS and ISBCS [9, 10]. A recent Cochrane systematic review from 2022 indicated no clinically important differences in outcomes between the two approaches. However, the outcomes were not compared considering ocular comorbidities and none of the included studies were conducted in low-income countries [3].

The Swedish National Cataract Register (NCR) was initiated in 1992 [11] and has amassed records for over 2.4 million cataract surgeries which are used for numerous scientific studies and quality improvements [12]. Detailed pre-, peri- and postoperative data are comprehensively described in previous publications [13, 14]. The social security number was introduced as a register variable in 2010, enabling a linkage between the first and second eye surgery and thereby the investigation into ISBCS and DSBCS. The register has good coverage among both public (92%) and private (95.4%) healthcare providers [15].

Our previous research, encompassing an ISBCS analysis, revealed a notable uptrend in ISBCS utilization in Sweden and that eyes undergoing ISBCS had less risk factors than eyes going through DSBCS [2]. Self-assessed benefit from ISBCS has been compared with DSBCS with 2 months between the first and second eye, showing significantly more self-reported difficulties in DSBCS until the second eye surgery was performed [5]. The benefit for the patient depends on the time between the first and second eye surgery as well as life expectancy [16].

Despite the advantage of ISBCS in bilateral cataract, the majority of cataract surgeries is performed as DSBCS, separated in time [3]. The rational for this is sometimes evident, such as good visual acuity in the other eye or risk factors such as corneal dystrophy, where an evaluation of the first eye surgery is recommended before the second eye surgery is performed [17]. Circumstances such as considerable visual impairment in both eyes due to cataract, planned multifocal IOL or expected anisometropia in one eye surgery are probable reasons for planning surgery in both eyes from the start, either with ISBCS or DSBCS. Our hypothesis was that additional factors influence the time between the first and second eye surgery, some of which can be seen as more unmotivated than others. The impact of the healthcare provider’s geographical location and whether it is a private or public entity has been shown to be associated with first and second eye surgery [2]. Patient characteristics such as age and sex as well as surgical complications and difficulties can also have an impact on DSBCS. In the current study, we investigated the variables that are associated with the time between the first and second eye surgery in DSBCS.

## Methods

This register-based cohort study compares NCR data from the 1st of January 2010 to the 31st of December 2019. This study solely relies on NCR data, where each cataract surgery was meticulously recorded by the surgeon or appointed personnel based on previous medical records and current surgery. To link the initial and subsequent eye surgeries of patients who underwent cataract surgery in both eyes during the study period, their records were linked using their unique social security number. Bilateral surgeries conducted on separate days were classified as DSBCS. This study followed the tenets of the Declaration of Helsinki and was approved by the Swedish Ethical Review Authority (2021–01532).

A binary outcome variable was established to denote whether a patient had bilateral cataract surgery within a 3-month period (≤ 3 months). Associations between surgery of the two eyes within 3 months and hypothesized predictors such as operation year, region, private or public surgical unit, age, sex, indication for surgery, type of intraocular lens (IOL), preoperative visual acuity, ocular comorbidity, posterior capsule rupture (PCR), and perioperative difficulties were analyzed with stratification and multivariate logistic regression. PCR was represented as a binary variable encompassing all instances of communication between the anterior and posterior chamber, inclusive of zonular damage. Included ocular comorbidities were age-related macular degeneration (AMD), diabetic retinopathy (DR), glaucoma, cornea guttata, uveitis, and pseudoexfoliation syndrome (PEX). Perioperative measures included were mechanical pupil dilation, trypan blue staining, capsular tension ring and capsule retractors.

Corrected distance visual acuity (CDVA) was reported to the register in decimals but was converted to logarithm of the minimum angle of resolution (logMAR) for analyses in the current study. Time between surgeries and preoperative CDVA were presented as median, and differences were tested using the Mann–Whitney U test. Differences between categorical variables were tested with the Chi-squared test. The Kaplan–Meier plot was used to estimate the time between first and second eye surgery, and the impact of second eye CDVA from the start. Differences with a *P* value less than 0.05 were considered statistically significant. The data were analyzed using RStudio version 1.4.1103 (RStudio Team, 2021, Boston, MA, USA).

## Results

A total of 1,110,281 cataract surgeries were registered in the NCR during the study period, 76% of which were performed in both eyes (n = 844,600). Consequently, the number of patients who underwent bilateral cataract surgery was 422,300, of which 13% (n = 54,194) had ISBCS that were performed on the same day.

**Fig. 1:**
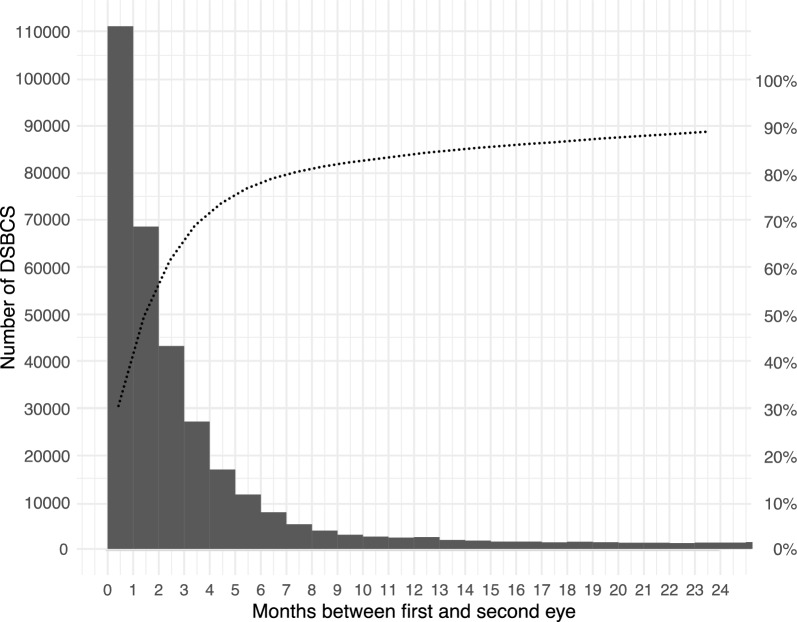
The pareto chart shows DSBCS with time between the first and second eye during the first two years. ISBCS are excluded. The curved dotted line shows the cumulative percentage of DSBCS. DSBCS, delayed sequential bilateral cataract surgery; ISBCS, immediate sequential bilateral cataract surgery

For DSBCS, the median interval between the first and second surgeries was 61 days [interquartile range (IQR) 26–161] and the monthly distribution of the second eye surgery is shown in Fig. 1. A majority of the DSBCS (63%, n = 230,331) received bilateral surgery within 3 months, 77% within 6 months and 84% within 1 year. The percentage of bilateral cataract surgeries completed within 3 months among bilateral operations within a year exhibited an ascending trend across each calendar year during the period studied, escalating from 64% in 2010 to 78% in 2018 (*P* < 0.001).

**Fig. 2:**
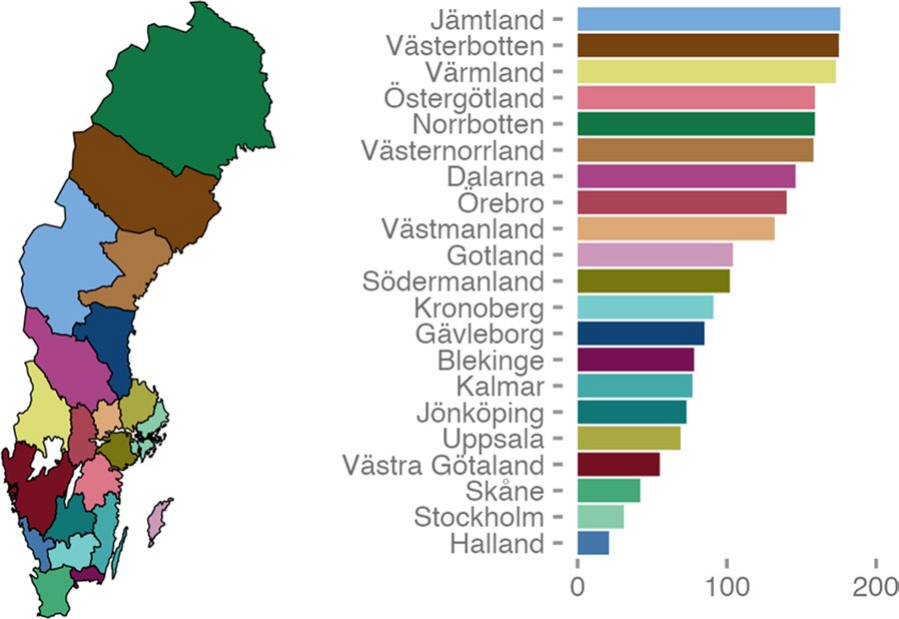
The median time (days) between first and second eye surgery in Sweden’s different healthcare regions during the study period

The surgeries for both the first and second eye was performed in the same Swedish healthcare region in 98% (n = 361,854) of the patients. The time between the surgery of the two eyes differed substantially between healthcare regions (Fig. 2). In the region with the shortest time (Halland), this interval was 21 days (IQR 9–57) in median compared to 176 days (IQR 101–718) in the region with the longest time (Jämtland) between the first and second eye surgery. In Halland, 61% (n = 8956) of the patients had cataract surgery in a private clinic. On the other hand, in Jämtland, all 2570 surgeries were performed in a public clinic.

A correlation was observed between the timing of the second eye surgery and whether the procedure took place in a private or public healthcare facility. In private clinics, the median duration was 33 days in median (IQR 14–82) compared to 86 days (IQR 41–238) in public clinics (*P* < 0.001). This signifies that 75% (third quartile) of the DSBCS in private clinics were completed within 82 days while the equivalent timeframe was 238 days for those in public clinics.

During the initial eye surgery, the median visual acuity in the second eye was 0.22 logMAR (IQR 0.15–0.40) (0.6 decimal) when the second eye surgery was performed within 3 months, compared to 0.19 logMAR (IQR 0.10–0.30) (0.65 decimal) when the second eye surgery was performed more than 3 months after the first surgery (*P* < 0.001).

**Fig. 3:**
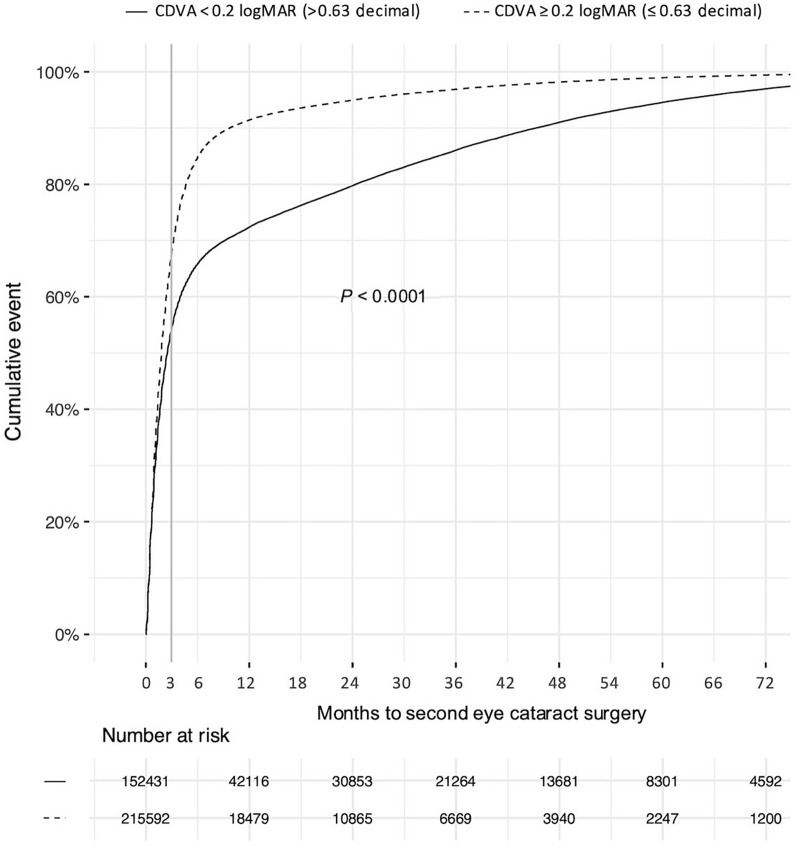
The Kaplan–Meier plot shows the cumulative distribution of DSBCS over time to second eye surgery. The groups are divided by second eye CDVA 0.2 logMAR (0.63 decimal) and worse at the time of the first eye surgery. DSBCS, delayed sequential bilateral cataract surgery; CDVA, corrected distance visual acuity

Among patients with CDVA 0.2 logMAR (0.63 decimal) or poorer in the second eye at the time of surgery of the first eye, 32% (n = 68,610) underwent surgery of the second eye after more than 3 months (Fig. 3). Half of these (n = 34,636) exhibited no ocular comorbidity and instances of PCR at the first eye surgery. Patients with better visual acuity (CDVA < 0.2 logMAR) in the second eye at the time for the first surgery waited longer with the second eye surgery (Fig. 3). An ocular comorbidity (AMD, DR, glaucoma, cornea guttata, uveitis, PEX) was reported in the first operated eye in 38% (n = 138,144). For those operated bilaterally within three months this proportion was 34% versus 43% for those with more than 3 months between the surgeries (*P* < 0.001). Ocular comorbidity in the first or second eye delayed bilateral surgery (Table 1).

**Table 1:** Table with overall data and stratified by DSBCS within or after three months

Variables	Overall	≤ 3 months DSBCS	> 3 months DSBCS	*P* value
	(n = 368,106)	(n = 230,311)	(n = 137,795)	
Sex, Female, n (%)	223,344 (60.7%)	141,340 (61.4%)	82,004 (59.5%)	< 0.001
Age, years median [IQR]	75 [69, 80]	75 [70, 80]	74 [68, 80]	< 0.001
Days between eyes, median [IQR]	61 [26, 161]	31 [14, 56]	273 [137, 910]	< 0.001
CDVA first eye, median logMAR [IQR]	0.40 [0.30, 0.60]	0.40 [0.30, 0.52]	0.40 [0.30, 0.70]	< 0.001
Missing, n (%)	184 (0.1%)	102 (0.0%)	82 (0.1%)	
CDVA second eye at first surgery, median logMAR [IQR]	0.22 [0.15, 0.40]	0.22 [0.15, 0.40]	0.19 [0.10, 0.30]	< 0.001
Missing, n (%)	78 (0.0%)	44 (0.0%)	34 (0.0%)	
Ocular comorbidity first eye, n (%)	138,144 (37.5%)	79,287 (34.4%)	58,857 (42.7%)	< 0.001
Glaucoma	30,500 (8.3%)	15,465 (6.7%)	15,035 (10.9%)	< 0.001
AMD	54,467 (14.8%)	34,049 (14.8%)	20,418 (14.8%)	0.78
DR	15,856 (4.3%)	7646 (3.3%)	8210 (6.0%)	< 0.001
Uveitis	144 (0.0%)	66 (0.0%)	78 (0.1%)	< 0.001
PEX	30,845 (8.4%)	17,873 (7.8%)	12,972 (9.4%)	< 0.001
Cornea guttata	9898 (2.7%)	5951 (2.6%)	3947 (2.9%)	< 0.001
Other	24,016 (6.5%)	13,181 (5.7%)	10,835 (7.9%)	< 0.001
Ocular comorbidity second eye, n (%)	126,785 (34.4%)	70,175 (30.5%)	56,610 (41.1%)	< 0.001
Glaucoma	27,573 (7.5%)	13,332 (5.8%)	14,241 (10.3%)	< 0.001
AMD	51,376 (14.0%)	31,329 (13.6%)	20,047 (14.5%)	< 0.001
DR	14,640 (4.0%)	6588 (2.9%)	8052 (5.8%)	< 0.001
Uveitis	233 (0.1%)	59 (0.0%)	174 (0.1%)	< 0.001
PEX	28,866 (7.8%)	14,588 (6.3%)	14,278 (10.4%)	< 0.001
Cornea guttata	9543 (2.6%)	5354 (2.3%)	4189 (3.0%)	< 0.001
Other	19,161 (5.2%)	10,578 (4.6%)	8583 (6.2%)	< 0.001
Perioperative measures, first eye, n (%)	26,734 (7.3%)	12,880 (5.6%)	13,854 (10.1%)	< 0.001
Perioperative measures, second eye, n (%)	20,549 (5.6%)	9238 (4.0%)	11,311 (8.2%)	< 0.001
Multifocal lens, n (%)	2515 (0.7%)	2277 (1.0%)	238 (0.2%)	< 0.001
Posterior capsule rupture first eye, n (%)	3145 (0.9%)	1122 (0.5%)	2023 (1.5%)	< 0.001
Posterior capsule rupture second eye, n (%)	2726 (0.7%)	1378 (0.6%)	1348 (1.0%)	< 0.001

Differences between DSBCS within or after 3 months were tested with Mann–Whitney U test for continuous variables and the Chi-Squared test for categorical variables

*DSBCS* = delayed sequential bilateral cataract surgery; *n* = number of participants; *IQR* = interquartile range; *CDVA* = corrected distance visual acuity; *AMD* = age-related macular degeneration; *DR* = diabetic retinopathy; *PEX* = pseudoexfoliation syndrome

Perioperative measures in the first eye surgery (mechanical pupil dilation, trypan blue staining, capsular tension ring, capsule retractors) were employed in 5.6% of the cases (n = 12,880) when the second eye surgery took place within 3 months compared to 10.1% (13,854) if the second eye surgery was performed later than after 3 months (*P* < 0.001; Table 1).

At the first eye surgery, 3145 patients had a PCR, of which 101 (3.2%) had a PCR in the second eye as well. PCR occurred in 0.5% (n = 1122) of first eye surgeries in DSBCS within 3 months. The corresponding proportion for those operated with more than 3 months between surgeries was 1.5% (*P* < 0.001). In other words, a complicated first eye surgery increased the time to surgery of the second eye.

Detailed data about indication for surgery were registered in the NCR between 2012 and 2017 (n = 272,649). Anisometropia was reported as indication for second eye surgery in 4.9% of all DSBCS cases, 5.7% (n = 13,099) within 3 months, and 3.7% (n = 5045) beyond 3 months (*P* < 0.001). This points to an association between anisometropia as the indication for the second eye surgery and a higher likelihood of undergoing bilateral surgery within 3 months. Increased intraocular pressure (IOP) as indication for surgery had no influence on the timing of bilateral cataract surgery. Conversely, the use of a multifocal IOL in the first eye operated was related to bilateral surgery within 3 months (*P* < 0.001; Table 1). Median time between the bilateral surgeries was 14 days (IQR 7–28) with a multifocal IOL in the first eye, compared to 62 days (IQR 27–162) without a multifocal IOL (*P* < 0.001).

The logistic regression analysis showed that the odds of having the second surgery within 3 months was lower in patients having good visual acuity (CDVA 0.1 logMAR or better), ocular comorbidity in the second eye, male sex, perioperative measures and posterior capsule rupture in the first eye (Table 2). Increasing age, year of surgery and private clinic increased the odds of having the second surgery within 3 months. Good vision in the fellow eye (OR = 0.33, *P* < 0.001) and private clinic (OR = 3.40, *P* < 0.001) had the strongest association with time to surgery of the second eye in the multivariate model (Table 2).

**Table 2:** Uni- and multivariate logistic regression models with DSBCS within 3 months as dependent variable

Variables	Unadjusted OR	95% CI	*P* value	Adjusted OR	95% CI	*P* value
Second eye CDVA ≤ 0.1 logMAR (0.8 decimal or better)	0.24	0.224–0.257	< 0.001	0.33	0.315–0.351	< 0.001
Age (years)	1.03	1.029–1.031	< 0.001	1.005	1.004–1.006	< 0.001
Sex, Male	0.86	0.844–0.876	< 0.001	0.96	0.948–0.98	< 0.001
Year of surgery	1.24	1.237–1.243	< 0.001	1.20	1.193–1.198	< 0.001
Posterior capsule rupture, first eye	0.39	0.317–0.459	< 0.001	0.42	0.337–0.501	< 0.001
Ocular comorbidity, second eye	0.61	0.595–0.626	< 0.001	0.63	0.612–0.644	< 0.001
Perioperative measures, first eye	0.58	0.551–0.604	< 0.001	0.66	0.634–0.691	< 0.001
Private clinic	2.50	2.482–2.518	< 0.001	3.40	3.379–3.412	< 0.001

Unadjusted OR was calculated for each variable with univariate logistic regression. The adjusted OR was calculated for each variable with multiple logistic regression with the other variables as confounders. *DSBCS* = delayed sequential bilateral cataract surgery; *OR* = odds ratio; *95% CI* = 95% confidence interval

## Discussion

Cataract is often a bilateral disease. This study examined factors associated with the first and second eye surgery in delayed sequential bilateral cataract surgery (DSBCS). The region where the surgery was performed had a substantial impact on the time between the surgeries from a median of 3 to 25 weeks. This is in line with previous studies, showing significant variations in regional healthcare practices within a single country [18]. Better visual acuity in the second eye, ocular comorbidity, various perioperative events and complications were associated with longer time to surgery of the second eye. Increasing age, cataract surgery in more recent years and in private clinics were associated with shorter timespan between the two surgeries.

Patients undergoing cataract surgery in public clinics experienced more than double the waiting time between surgery of both eyes compared to private clinics (86 versus 33 days in median). The number and proportion of cataract surgeries performed in private clinics with public financing has seen a yearly rise in Sweden over the past decade, reaching 63% in 2021 [12]. This trend may have contributed to a decreased duration before the second eye surgery over the study period although the multivariate model suggests that this progression was influenced by additional factors.

In certain scenarios it is clear why a shorter time between surgeries in DSBCS might be warranted. For instance, when a multifocal IOL is used in the first eye or in case of induced anisometropia, surgery is often planned for both eyes from the start.

A variable indicating whether a patient had bilateral cataract surgery within 3 months or not was created, assuming that the indication for bilateral surgery was present from the beginning in DSBCS within 3 months. A Swedish national minimum waiting time guarantee for healthcare was operative during the study period, and states that the patient has the right to surgery within 90 days (i.e., 3 months) following the decision for the procedure. This guarantee is applicable to scheduled surgery and not DSBCS. In 2019, 88% of all cases were operated within a waiting time of 3 months i.e., almost all cases had surgery within 3 months if the second eye was planned at the surgery of the first eye [15]. The objective of the 90-day guarantee is to minimize and manage waiting times effectively, ensuring timely healthcare delivery. It is important to consider the patient's overall visual function comprehensively, and that each eye is not handled completely separately just to fulfill the guarantee.

A substantial proportion of the patients (32%) underwent the second eye surgery with an interval of more than 3 months despite a visual impairment in the second eye (CDVA ≥ 0.2 logMAR) at the planning of the first eye surgery (Fig. 3). Among these patients, many did not manifest ocular comorbidity or a posterior capsule rupture during surgery of the first eye and probably had indication for surgery of the second eye right from the start. A number of patients in this group would probably benefit from a less delayed second eye surgery. ISBCS would even be preferred in many cases, as it has been shown to have many benefits such as lower cost and efficacious in reducing the growing backlog of treatable blindness with faster visual recovery [8, 19]. Many patients have waited several months for the first visit to the ophthalmologist and additional months for the first eye surgery. It is of value for the patient to keep the waiting time between the first and second eye surgery short as the self-reported visual function improves significantly when both eyes are operated [16]. The number of cataract surgeries in Sweden has steadily increased in recent decades and it is important that there is a symptomatic visual impairment or medical indication for surgery [12]. According to Swedish national guidelines, great restrictiveness applies to cataract surgery in the case of visual acuity above 0.7 decimal and lack of medical indication [20].

The strength of this study, but at the same time a challenge, is the large number of included patients. It gives a high statistical power in all analyses, where small group differences and effects were possible to detect. For example, there was a statistically significant difference in preoperative visual acuity between DSBCS within and after 3 months even though the median CDVA was 0.4 logMAR in both groups. This highlights the importance to evaluate the effect size and its clinical relevance.

Another strength is NCR's good coverage throughout Sweden and the study's long duration of a decade. It enables a cohesive analysis of DSBCS and the changes over time in a whole country. Bias caused by local and regional differences is reduced with the national approach. The risk of selection bias is considered limited based on the fact the data includes all cataract surgeries performed in a whole nation over a ten-year period from a register with 94% coverage.

One limitation of this study is its reliance on a register-based study design, which may overlook certain factors. For instance, the register does not capture if there was a symptomatic cataract in the other eye at the time of first eye surgery or not. Moreover, other ocular surgeries that potentially delayed the second eye cataract surgery e.g., vitrectomy or keratoplasty, would be unknown to this study as it is solely based on the NCR [21–24]. Another limitation is the fact that some DSBCS cases might have been planned as ISBCS, but changed to DSBCS at the time for surgery e.g., due to PCR. With the available data from the NCR, it was not possible to distinguish these cases from those in whom DSBCS was the intended treatment plan from the beginning. In the example of PCR, however, it would affect the study in the same direction that the results point towards. PCR, ocular comorbidity and perioperative difficulties prolonged the time between first and second eye surgery. Further studies on differences in the Swedish healthcare are warranted, especially regarding regional differences and the impact of health care provider (private or public). The association between socioeconomic status and the time between first and second eye cataract surgery is also recommended to be studied further to provide additional insights in this area.

## Conclusions

The vast majority of DSBCS were performed with a few months apart and this study has shown variables associated with the time between the surgeries. While some of these factors are logical, others may seem less so. As many patients would benefit from less time between the surgeries, we encourage a clinical practice taking the patient’s whole visual function into account during the planning of the first eye surgery.

## Data Availability

The datasets analyzed during the current study are not publicly available due to personal data protection policies. The dataset is from The Swedish National Cataract Register.

## Abbreviations

DSBCS: Delayed sequential bilateral cataract surgery
NCR: National Cataract Register
IOL: Intraocular lens
IQR: Interquartile range
OECD: Organization for Economic Cooperation and Development
AMD: Age-related macular degeneration
DR: Diabetic retinopathy
PEX: Pseudoexfoliation syndrome
CDVA: Corrected distance visual acuity
logMAR: Logarithm of the minimum angle of resolution
PCR: Posterior capsule rupture
IOP: Intraocular pressure
OR: Odds ratio

## References

[1] Flaxman SR, Bourne RRA, Resnikoff S, Ackland P, Braithwaite T, Cicinelli MV, et al. Global causes of blindness and distance vision impairment 1990–2020: a systematic review and meta-analysis. Lancet Glob Health. 2017;5(12):e1221–34. 29032195 10.1016/S2214-109X(17)30393-5

[2] Lundström M, Kugelberg M, Zetterberg M, Nilsson I, Viberg A, Bro T, et al. Ten-year trends of immediate sequential bilateral cataract surgery (ISBCS) as reflected in the Swedish National Cataract Register. Acta Ophthalmol. 2024;102(1):68–73. 37133405 10.1111/aos.15688

[3] Dickman MM, Spekreijse LS, Winkens B, Schouten JS, Simons RW, Dirksen CD, et al. Immediate sequential bilateral surgery versus delayed sequential bilateral surgery for cataracts. Cochrane Database Syst Rev. 2022;4(4):CD013270. 35467755 10.1002/14651858.CD013270.pub2 PMC9037598

[4] Leivo T, Sarikkola AU, Uusitalo RJ, Hellstedt T, Ess SL, Kivelä T. Simultaneous bilateral cataract surgery: economic analysis; Helsinki Simultaneous Bilateral Cataract Surgery Study Report 2. J Cataract Refract Surg. 2011;37(6):1003–8. 21596243 10.1016/j.jcrs.2010.12.050

[5] Lundström M, Albrecht S, Nilsson M, Aström B. Benefit to patients of bilateral same-day cataract extraction: randomized clinical study. J Cataract Refract Surg. 2006;32(5):826–30. 16765801 10.1016/j.jcrs.2006.01.075

[6] Limburg H, Ramke J. Cataract indicators: their development and use over the last 30 years. Community Eye Health. 2017;30(100):82–4. 29483752 PMC5820632

[7] OECD. OECD Health Statistics 2023 Definitions, Sources and Methods. 2023. https://stats.oecd.org/fileview2.aspx?IDFile=53847866-4eb2-4238-84d4-f5470ad4ffba. Accessed 1 Feb 2024.

[8] Singh G, Grzybowski A. Evolution of and developments in simultaneous bilateral cataract surgery. Update 2020. Ann Transl Med. 2020;8(22):1554. 33313299 10.21037/atm-20-3490 PMC7729368

[9] Herrinton LJ, Liu L, Alexeeff S, Carolan J, Shorstein NH. Immediate sequential vs. delayed sequential bilateral cataract surgery: retrospective comparison of postoperative visual outcomes. Ophthalmology. 2017;124(8):1126–35. 28438415 10.1016/j.ophtha.2017.03.034 PMC5531866

[10] Lacy M, Kung TH, Owen JP, Yanagihara RT, Blazes M, Pershing S, et al. Endophthalmitis rate in immediately sequential versus delayed sequential bilateral cataract surgery within the Intelligent Research in Sight (IRIS**®**) registry data. Ophthalmology. 2022;129(2):129–38. 34265315 10.1016/j.ophtha.2021.07.008 PMC8755857

[11] Stenevi U, Lundström M, Thorburn W. A National Cataract Register. I. Description and epidemiology. Acta Ophthalmol Scand. 1995;73(1):41–4. 7627757 10.1111/j.1600-0420.1995.tb00011.x

[12] Bro T, Behndig A, Viberg A, Zetterberg M, Kugelberg M, Nilsson I, et al. Two point four million cataract surgeries: 30 years with the Swedish National Cataract Register, 1992–2021. J Cataract Refract Surg. 2023;49(8):879–84. 37185666 10.1097/j.jcrs.0000000000001209

[13] Behndig A, Montan P, Stenevi U, Kugelberg M, Lundström M. One million cataract surgeries: Swedish National Cataract Register 1992–2009. J Cataract Refract Surg. 2011;37(8):1539–45. 21782099 10.1016/j.jcrs.2011.05.021

[14] Zetterberg M, Montan P, Kugelberg M, Nilsson I, Lundstrom M, Behndig A. Cataract surgery volumes and complications per surgeon and clinical unit: data from the Swedish National Cataract Register 2007 to 2016. Ophthalmology. 2020;127(3):305–14. 31767438 10.1016/j.ophtha.2019.10.007

[15] Svensk Kataraktkirurgi - Årsrapport 2019 baserad på data från Nationella Kataraktregistret, https://kataraktreg.se/publikationer/arsrapporter. Accessed 1 Sept 2024.

[16] Lundström M, Albrecht S, Roos P. Immediate versus delayed sequential bilateral cataract surgery: an analysis of costs and patient value. Acta Ophthalmol. 2009;87(1):33–8. 18786128 10.1111/j.1755-3768.2008.01343.x

[17] Viberg A, Samolov B, Claesson Armitage M, Behndig A, Byström B. Incidence of corneal transplantation after phacoemulsification in patients with corneal guttata: a registry-based cohort study. J Cataract Refract Surg. 2020;46(7):961–6. 32271268 10.1097/j.jcrs.0000000000000207

[18] Corallo AN, Croxford R, Goodman DC, Bryan EL, Srivastava D, Stukel TA. A systematic review of medical practice variation in OECD countries. Health Policy. 2014;114(1):5–14. 24054709 10.1016/j.healthpol.2013.08.002

[19] O’Brien JJ, Gonder J, Botz C, Chow KY, Arshinoff SA. Immediately sequential bilateral cataract surgery versus delayed sequential bilateral cataract surgery: potential hospital cost savings. Can J Ophthalmol. 2010;45(6):596–601. 21135895 10.3129/i10-094

[20] NPO-ögonsjukdomar. Riktlinje för katarakt 2022, https://swedeye.org/wp-content/uploads/2022/01/Riktlinje-for-katarakt-220114.pdf. Accessed 1 Feb 2024.

[21] Rohowetz LJ, Jabbehdari S, Yannuzzi NA, Sridhar J, Smiddy WE, Berrocal AM, et al. Pars plana vitrectomy for retained lens fragments after cataract surgery: outcomes based on timing of surgery. Clin Ophthalmol. 2023;17:479–85. 36755889 10.2147/OPTH.S391795 PMC9899933

[22] Matarazzo F, Phylactou M, Aiello F, Gallo Afflitto G, Yue Sim S, Maurino V. Incidence and complications of retained lens fragment in the anterior chamber after uneventful cataract surgery in a United Kingdom tertiary center. J Cataract Refract Surg. 2021;47(8):1064–70. 34292892 10.1097/j.jcrs.0000000000000585

[23] Zavodni ZJ, Meyer JJ, Kim T. Clinical features and outcomes of retained lens fragments in the anterior chamber after phacoemulsification. Am J Ophthalmol. 2015;160(6):1171–5.e1. 26299538 10.1016/j.ajo.2015.08.019

[24] Modi YS, Epstein A, Smiddy WE, Murray TG, Feuer W, Flynn HW Jr. Retained lens fragments after cataract surgery: outcomes of same-day versus later pars plana vitrectomy. Am J Ophthalmol. 2013;156(3):454–9.e1. 23810473 10.1016/j.ajo.2013.04.038

